# The Stepping Stones and Creating Futures intervention to prevent intimate partner violence and HIV-risk behaviours in Durban, South Africa: study protocol for a cluster randomized control trial, and baseline characteristics

**DOI:** 10.1186/s12889-017-4223-x

**Published:** 2017-04-20

**Authors:** Andrew Gibbs, Laura Washington, Samantha Willan, Nolwazi Ntini, Thobani Khumalo, Nompumelelo Mbatha, Yandisa Sikweyiya, Nwabisa Shai, Esnat Chirwa, Michael Strauss, Giulia Ferrari, Rachel Jewkes

**Affiliations:** 1 Gender and Health Research Unit, South African Medical Research Council, Pretoria, South Africa; 2 Health Economics and HIV/AIDS Research Division (HEARD), University of KwaZulu-Natal, Durban, South Africa; 3 Project Empower, Durban, South Africa; 4 London School of Hygiene and Tropical Medicine, London, UK

## Abstract

**Background:**

Preventing intimate partner violence (IPV) remains a global public health challenge. Studies suggest urban informal settlements have particularly high levels of IPV and HIV-prevalence and these settlements are rapidly growing. The current evidence base of effective approaches to preventing IPV recognizes the potential of combining economic strengthening and gender transformative interventions. However, few of these interventions have been done in urban informal settlements, and almost none have included men as direct recipients of these interventions.

**Methods:**

Stepping Stones and Creating Futures intervention is a participatory gender transformative and livelihoods strengthening intervention. It is being evaluated through a cluster randomized control trial amongst young women and men (18–30) living in urban informal settlements in eThekwini Municipality, South Africa. The evaluation includes a qualitative process evaluation and cost-effectiveness analysis. A comparison of baseline characteristics of participants is also included.

**Discussion:**

This is one of the first large trials to prevent IPV and HIV-vulnerability amongst young women and men in urban informal settlements. Given the mixed methods evaluation, the results of this trial have the ability to develop a stronger understanding of what works to prevent violence against women and the processes of change in interventions.

**Trial registration:**

NCT03022370. Registered 13 January 2017, retrospectively registered.

**Electronic supplementary material:**

The online version of this article (doi:10.1186/s12889-017-4223-x) contains supplementary material, which is available to authorized users.

## Background and rationale

Global statistics indicate high levels of women’s victimization by intimate and non-partners, with an estimated 36% of women globally having experienced physical and/or sexual intimate partner violence (IPV) or non-partner sexual violence in their lifetime [1]. In South Africa, violence and injuries are the second leading cause of death and loss of disability-adjusted life years [2]. Population-based estimates for South Africa from 2010, show a lifetime prevalence in adult women of physical IPV victimisation of 33% and past-year prevalence of 13%, and 40% of men disclose having perpetrated physical IPV [3]. A quarter of women have been raped by a partner or non-partner, and between 28 and 37% of men disclose rape perpetration of partner or non-partner in surveys [3, 4].

Women’s experiences of IPV, alongside violating their human rights, constitute a key health burden. Studies suggest women who experience physical and/or sexual IPV are more likely to be depressed and suicidal [5], consume higher levels of alcohol [6], have greater numbers of unplanned pregnancies, and increased induced abortions [1]. In addition, in southern and eastern Africa they are between 15 and 25% more likely to acquire HIV [7].

Urban informal settlements, globally and in South Africa, are rapidly expanding [8]. These are spaces with a high prevalence of major health problems, including HIV, and IPV, which particularly affect young people [8–11]. In South Africa HIV-prevalence in informal settlements is twice that of formal housing settlements [12, 13], and IPV-incidence among young people (18–30) is between 3 and 5 times national estimates [14].

A range of theories explain the high levels of IPV and HIV in southern and eastern Africa, and particularly urban informal settlements. One body of research links poverty and material inequality to HIV and IPV risk [15, 16]. Others emphasise mobility and the weak social relationships existing in urban informal settlements, undermining social forms of power that have a tendency to constrain certain behaviours [17]. A cross-cutting explanation are the ways in which gender inequalities, particularly in contexts of poverty, are pronounced. This combination places women in economically and socially dependent relationships with men, and thus at higher risk of experiencing IPV and HIV-vulnerability [15, 18]. For men, it is argued their experience of economic marginalisation limits them from achieving respectability and a sense of masculine success through providing for their household, a key feature of masculinity in many communities. In turn they seek other forms of identity and respect, namely through control and dominance over women sexually and physically [15, 19, 20].

### Current evidence on gender transformative plus economic strengthening interventions

Current evidence around interventions to reduce women’s experiences of IPV and HIV-vulnerability are located in gender transformative approaches, whether working with women or with men [21, 22]. As Ellsberg and colleagues [21] comment, these approaches “address underlying expectations about male and female roles and behavior…through a process of critical reflection, discussion, and practice.” There remains a paucity of well-evaluated group-based, gender transformative interventions. One of the few interventions showing effect was the Stepping Stones RCT, implemented in the rural Eastern Cape of South Africa. At 12 months follow-up, men reported less transactional sex with a casual partner, and less problematic alcohol use. At 24 months, men reported less perpetration of sexual and/or physical IPV [23]. Other group-based interventions have shown promise at reducing IPV but have often been limited by small sample sizes. In Cote d’Ivoire, for instance, a group-based intervention for men, seemed to reduce men’s perpetration of IPV, but the effect was not significant [24].

More evaluations have shown promise through combining group-based gender transformative interventions with economic strengthening interventions for women [21, 25]. The IMAGE study combined micro-finance training with a gender transformative component and community mobilization for women in rural South Africa. This showed a 55% significant reduction in IPV experienced by women who participated in the intervention [26]. In addition, the IMAGE study reported positive changes around HIV-risk behaviours for young women (under 35), including an increase in condom use at last sex with non-cohabiting partners, and greater communication about HIV [27]. Similar interventions have shown positive outcomes, but often effects have not been significant [28].

Translating economic empowerment and gender transformative interventions into approaches for young women initially proved challenging. An early generation of interventions including SHAZ! – which combined microfinance and gender training - and TRY showed promise, but no significant results in terms of reducing IPV [25]. More recently, Bandiera and colleagues report on a combined microfinance, life-skills training and vocational training in Uganda which reduced young women’s experiences of sexual violence significantly [29]. While in Zimbabwe, SHAZ! was adapted to respond to the challenges in the pilot, and included vocational training, a small non-repayable grant and gender training. After 24 months, women reported a reduction in IPV and HIV-risk behaviours [30].

The majority of the gender transformative and economic strengthening interventions have focused on working directly with women. Where men have been included, it has tended to be partial inclusion, often as the woman’s husband/partner [25]. Only one pilot study in the US explicitly included young men in a combined economic and gender transformative intervention, and reported a range of positive outcomes, but could not look at IPV perpetration as the sample was too small [31].

The lack of rigorous evaluations of men involved in gender transformative and economic strengthening interventions is problematic. First, many interventions are working to strengthen men’s employment and livelihoods, without an analysis of the impact of these on IPV. Second, there may be benefits of including men in such interventions. A central argument about men’s perpetration of IPV is that violence emerges when men are structurally excluded from economic opportunities and then use violence as a way to achieve some form of respect [19, 32]. On the other hand, some research has shown men who are poor, but not the poorest, may have higher rates of perpetration of violence against women (VAW) [4, 33], so understanding how men respond to interventions of this type is very important.

This paper describes the Stepping Stones and Creating Futures Intervention trial in terms of the SPIRIT (Additional file 1: Standard Protocol Items: Recommendations for Interventional Trials) 2013 Checklist, alongside a comparison of baseline characteristics of participants. The trial is a cluster randomized control trial that seeks to assess the effectiveness of Stepping Stones and Creating Futures in reducing IPV and HIV-risk behaviours and strengthening livelihoods. The evaluation has two arms, with the intervention arm receiving the Stepping Stones and Creating Futures intervention, while the control arm are wait-listed. Follow-up for primary and secondary outcomes are at 24 months.

The trial is a collaborative effort between the South African Medical Research Council and the Health Economics and HIV/AIDS Research Division (HEARD) at the University of KwaZulu-Natal who are undertaking the quantitative and qualitative evaluation. Project Empower a gender and HIV non-governmental organization based in Durban, South Africa and experienced in working and delivering a range of interventions in informal settlements and in implementing programmes within the parameters of research projects. The London School of Hygiene and Tropical Medicine (LSHTM), are leading the cost-effectiveness evaluation in collaboration with the team.

The trial is being conducted as part of the portfolio of research of the What Works To Prevent Violence? A Global Programme on Violence Against Women and Girls (VAWG) funded by the UK Government’s Department for International Development (DFID).^1^ The Global Programme funding research into 17 interventions in 14 countries to assess the effectiveness of the interventions in prevention of VAWG. The work is being conducted in Africa, the Middle East and Asia, between 2014 and 2018.

## Methods

### Objectives

The main objectives of the trial are to determine through a cluster randomized control trial (RCT) whether the combined Stepping Stones and Creating Futures interventions are effective in enabling young women and men (18–30) in informal settlements to reduce their exposure/perpetration to physical and/or sexual IPV and strengthen young women and men’s livelihoods.

### Trial design

The trial is a two-arm cluster randomized control trial, with 12 month and 24 month follow-up. Intervention clusters receive Stepping Stones and Creating Futures immediately, while control arm clusters are wait-listed to receive the intervention at end of follow-up.

### Methods: Participants, interventions and outcomes

#### Study setting

Urban informal settlements in eThekwini municipality, KwaZulu-Natal, the third largest city in South Africa. In eThekwini municipality an estimated 25% of the population live in informal housing [34]. Informal settlements are characterised by dense housing, and poor access to water and electricity.

#### Eligibility criteria

At the cluster level, eligibility is defined as urban informal settlements in the eThekwini Municipality, South Africa in communities where Project Empower, the implementing partner, have assessed it is safe to work. Informality is defined as not having formal service provision within the home, such as no electricity legally supplied and no piped water to housing.

Defining an informal settlement as a cluster is not straightforward. In cluster randomized trials, there are often clear governmental boundaries that delineate clusters [35]. For instance, in SASA! clusters were defined as municipal parishes [36], while in IMAGE, rural villages could be easily identified [27]. Informal settlements do not follow governmental boundaries. Rather, they are located on previously under-used land, steep hillsides, or areas prone to flooding, and fill spaces between formal housing.

Despite these challenges, larger informal settlements in eThekwini are typically geographically bounded. The significant variation in size of informal settlements is dealt with in three ways to create comparable clusters: 1) Small informal settlements close to each other were clustered together to form a larger informal settlement cluster where we can recruit participants; 2) Large informal settlements, were split into separate clusters using naturally occurring boundaries and space to limit the potential for contamination; and, 3) In analyses we will account for variation in baseline characteristics of clusters.

Eligible participants must report being normally resident in an informal settlement, aged 18–30 years, not in formal employment, able to communicate in the main languages of the study (English, isiXhosa or isiZulu) and not suffering from a learning difficulty, or be currently psychotic, drunk or drugged, which would impair their ability to consent to participation in the trial.

#### Intervention

The intervention comprised two separate intervention manuals, the third edition of the South African adaptation of Alice Welbourn’s Stepping Stones [37] and Creating Futures [38]. The rationale for the combined intervention is shown in the Theory of Change for the combined interventions (Table 1).

**Table 1:** Stepping Stones and Creating Futures theory of change

Interventions	Creating futures		Stepping stones					
Overall result	REDUCED VAWG AND OTHER OUTCOMES							
Hypothesised effect	Greater engagement in income generating activity gives women more social power and enhances men’s selfworth		Critical reflection methodology challenges acceptance of patriarchy, opens doors to more respectful masculinities and more assertive femininities, all leading to less VAWG			Empowerment overall and better relationships enhance mood and reduces substance abuse		Empowering methodology and group work combined with communication skills improves relationships and handling of disagreements
Required intervention elements	Life skills intervention to assist income generation or return to education		Focus on building and understanding of gender equity and tackling VAWG					Building communication skills combined with gender equity
Amenable risk and aggrevating factors	Low economic power	Low education	Masculinities predicated on dominance over & control of women	Women’s acceptance of patriarchal dominance	Ingrained acceptability of use of VAWG	Depression	Men’s substance abuse	Poor relationship skills (esp.over conflict)
MEN AND WOMEN								
Problem statement	One third of South African women experience VAWG in their lifetime and this is higher in some local areas. The problem is driven substantially by the low status and power of girls and women, and social norms related to masculinity which emphasise dominance and control over women. In informal settlements the situation is exacerbated by very high youth unemployment and generally low levels of education (nationally 60% of learners fail to reach matric).							

This edition of Stepping Stones has ten, three-hour sessions delivered to single-sex groups of about 14–20 participants. It spans 30 h and is run over 6–8 weeks. It uses participatory learning approaches, including critical reflection, role play and drama and draws the everyday reality of participants’ lives into the sessions. The content includes: how we act and what shapes it (gender and peer influences); sex and love; conception and contraception; STIs and HIV; safer sex and condoms; gender-based violence; motivations for sexual behavior (including alcohol and poverty); and communication skills. Our South African adaptation has slightly different content from the Welbourn [39] original. It places a greater emphasis on sexual health and gender-based violence, and in this trial is run without peer group meetings. Welbourn [39] recommended working with older men and women in each community as well as youth, and suggested that peer groups be encouraged to continue to meet after the end of the workshops. We are not doing either, as it would add greatly to the cost of the intervention, which is already made more complex by the combination with Creating Futures. Further, our design deliberately focuses on changes and relationships within and among young people, and while recognizing the existence of intergenerational relationships, South African communities have very important age/sex group peer influences and we did not feel the focus on the older generation was the correct focus for this study. Additionally, our previous research in the rural Eastern Cape Province of South Africa has shown that Stepping Stones works effectively with young people to reduce sexual risk-taking and men’s use of IPV outside a community development context [23].

Creating Futures has eleven, three-hour sessions that are delivered immediately following the Stepping Stones sessions to the same single-sex groups. Overall contact time is approximately 33 h. Key issues covered include setting livelihood goals, coping with crises, saving and spending, getting and keeping jobs, and managing work expectations. There is also a significant focus on small-income generating activities [38]. Creating Futures is located within a sustainable livelihoods framework, emphasising that people survive through drawing on five capitals: financial, human, social, physical and natural [40]. Conventionally, approaches to addressing poverty include interventions such as micro-loan schemes, and technical or business training. Creating Futures treats these interventions as resources, which may or may not be available in participants’ contexts. Rather than offering a single resource as a stand-alone remedy for livelihoods strengthening, the curriculum engages participants in exercises which enable them to evaluate all available capitals and to map these into a single implementable livelihoods strategy.

#### Pilot research and field testing

The Stepping Stones and Creating Futures combined intervention was piloted among young adults in eThekwini informal settlements in 2012–13. 123 women and 110 men were recruited and received the intervention and then followed up 6 and 12 months later. Retention in the study at 12 months was 88%. Data suggest that average attendance at sessions was approximately 60% [14].

Pilot results from long-term qualitative and quantitative research have been published [14, 20, 41]. Analysis showed men’s mean earnings in the past month increased by 247% from R411 (~$40) to R1015 (~$102), and women’s by 278% R 174 (~$17) to R 484 (about $48) (trend test, *p* < 0.0001). There was a significant reduction in women’s experience of the combined measure of physical and/or sexual IPV in the prior three months from 30.3% to 18.9% (*p* = 0.037). Both men and women scored significantly better on gender attitudes, and men significantly reduced controlling practices in their relationships. Prevalence of moderate or severe depression symptomatology and suicidal thoughts among men decreased significantly (*p* < 0.0001; *p* = 0.01 respectively) [14]. Qualitative data from the pilot study suggested that women and men enjoyed participation in the intervention [20, 41]. In qualitative research primarily focused on men, men reported increased livelihood opportunities and greater savings [20].

#### Trial outcomes

Reflecting the multi-component approach of Stepping Stones and Creating Futures and the expected multiplicity of impacts, and building on recent approaches to evaluating complex interventions [36, 42], five primary outcomes, in two groups, reflecting the objectives of the study have been identified. Four primary outcomes are linked to IPV:

- Any past year physical IPV perpetration (men), and experience (women). This is assessed using a modified WHO VAW scale, that has been adapted and widely used in South Africa [7]. Five questions are asked about physical IPV perpetration (men) and experience (women) in the past 12 months. Past year physical IPV is coded as positive (1) for anyone responding positively to one or more items on the scale;
- Any past year sexual IPV perpetration (men), and experience (women) uses the same approach as for physical IPV. Three sexual IPV questions are asked about experiences in the past 12 months. Past year sexual IPV is coded as positive (1) for anyone responding positively to one or more items on the scale;
- To assess severe past year sexual and/or physical IPV perpetration (men) and experience (women), physical and sexual IPV scales are combined to be a total of eight items. Past year severe sexual and/or physical IPV is assessed as positive if a person responds to two (or more) items once, or one item as few (or more), essentially creating a more than once categorization. This approach follows previous studies [7].
- Controlling behaviours are assessed using a modified Sexual Relationship Power (SRP) scale [43] with 8 items. Higher scores refer to more controlling behaviours;

One primary outcome reflects the objective of the Creating Futures component of the intervention, around strengthening livelihoods:

- Past month earnings are used to assess overall income and livelihoods. A single item question asks “Considering all the money you earned from jobs or selling things (excluding grants), how much did you earn last month?” Responses are in South African Rands (ZAR) and a continuous scale.

We recognise it is conventional to keep the number of primary outcomes to the lowest possible number. However, there is no agreed binary goal in VAW research, that would be equivalent to e.g. acquiring or not acquiring HIV. VAW has different manifestations (we consider here physical and sexual violence, and controlling behaviours which are a dimension of emotional violence) and forms have different severities. It is not agreed whether it is more important to stop all women having any exposure or to reduce severe exposure, and nor is it agreed whether sexual violence is more or less important than physical violence or controlling behaviours. Research in South Africa on HIV and violence, for example, has shown that controlling behaviours and more severe physical and/or sexual violence are the exposures more strongly predictive of health risk [43]. In the absence of clear agreement on this we are measuring multiple dimensions of violence and consider all would be important if found to show difference between arms in the trial.

#### Secondary outcomes

Similar to the primary outcomes, five groups of secondary outcomes are identified focused on pathways to change through which the intervention is hypothesised to operate.

The first pathway reflects gender attitudes and norms in the group:

- Gender attitudes are assessed using a modified Gender Equitable Men’s Scale (GEMS) [44] adapted and widely used in South Africa [18]. The scale comprises of 20 questions, with larger scores indicating less gender equitable attitudes. It is hypothesised the mean will decrease.

Three sets of questions assess mental health and wellbeing:

- Past week depressive symptomatology is assessed by the Centre for Epidemiologic Studies Depression (CESD) scale, with the full twenty items [45]. Higher scores indicate greater depressive symptomatology. A mean score for each participant will be calculated. It is hypothesised the mean score will reduce.
- Past four-week suicidal ideation is assessed using a single item question and a binary yes/no response. It is hypothesised that the percentage of participants reporting yes to this will decrease.
- Life circumstances are assessed using four items derived from the Satisfaction With Life Scale [46]. Higher scores indicate greater life satisfaction. It is hypothesised the mean score will increase.

Two questions assess the impact of the intervention on alcohol use amongst participants as secondary outcomes:

- Problem drinking in the past year is assessed using the ten item Alcohol Use Disorders Identification Test (AUDIT) scale. Problem alcohol use is classified as respondents scoring 8 points or more [47]. It is hypothesised the percentage reporting problematic alcohol use will decrease.
- A single item assesses quarrelling in the past year about alcohol consumption with a sexual partner, assessed with a binary yes/no response. The intervention assumes the percentage reporting quarrelling about alcohol use will decrease.

Two items assess sexual risk behaviour.

- A single item assesses who the participant last had sex with. Response options are: “*main partner, kwapheni (casual) partner, once-off sex partner, ex-partner*.” Responses will be coded into a binary of main partner (1) or other sex partner (0). It is hypothesised that the percentage of participants reporting last sex with a main partner will increase.
- Transactional sex with a *kwapheni* or once-off sexual partner in the past year will be assessed using a five item scale used widely in South Africa [48]. A positive response to at least one of these items is classified as responding positively to transactional sex in the past 12 months. It is hypothesised this will reduce.

Livelihoods are assessed using four scales assessing material outcomes and psychological outcomes of limited income and work opportunities.

- Shame about lack of work is assessed using four items drawn from the IMAGES study [49]. Higher scores indicate greater levels of shame about lack of work and income. A mean score will be calculated for each participant and it is hypothesised the score will reduce.
- Stress related to lack of work and income is assessed on a four item scale drawn from the IMAGES study [49]. Higher scores indicate higher levels of stress about lack of work. A mean score will be calculated for each participant and it is hypothesised the score will reduce.
- Ability to mobilise cash in an emergency will be assessed with a single item. For analysis a binary will be created through collapsing very difficult and somewhat difficult to indicate challenges (and coded 0), while fairly easy and easy will be coded as no challenge (1). It is hypothesised the percentage of respondents reporting it is fairly easy or easy will increase.
- Stealing in the past four weeks because of hunger or lack of money will be assessed with a single item. Responses are: *Never, once, two or three times, more often*. Once, two or three times and more often will be collapsed together for analysis as indicating stealing because of hunger or lack of money. It is hypothesised this will decrease.

Table 2 below summarises the primary and secondary outcomes.

**Table 2:** Primary and secondary outcomes for the trial

	Typical item	Response categories	Number of items	Women’s cronbach alpha	Men’s cronbach alpha	Method of scaling	Hypothesised direction
Primary outcomes							
Physical IPV at 24 months	In the last 12 months how many times did you push or shove your current or previous girlfriend or wife?	Never, once, few many	5			Binary - never compared to once or more	Decrease
Sexual IPV at 24 months	In the last 12 months, how many times have you ever forced your current or previous girlfriend or wife to do something sexual that she did not want to do?	Never, once, few many	3			Binary - never compared to once or more	Decrease
Severe IPV at 24 months	As above	Never, once, few many	8			Binary: never or once, compared to more than once	Decrease
Controlling Behaviours at 24 months	I want to know where my partner is all of the time.	Four point Likert: Strongly disagree, disagree, agree, strongly agree	8	0.75	0.68	Mean	Decrease
Earnings in the past month at 24 months	Considering all the money you earned from jobs or selling things, how much did you earn in the last 4 weeks (not including grants)?	Number	1			Mean	Increase
Secondary outcomes							
Gender attitudes at 24 months	I think that a woman needs her husband’s permission to do paid work	Four point Likert: Strongly disagree, disagree, agree, strongly agree	20	0.86	0.86	Mean	Decrease
Depressive symptomology (CESD) at 24 months	During the past week I thought my life had been a failure	Rarely or none of the time; some or a little of the time; moderate amount of time; most or all of the time	20	0.88	0.87	Mean	Decrease
Suicidal Ideation at 24 months	In the past four weeks, has the thought of ending your life been in your mind?	Binary: Yes/No	1			Binary	Decrease
Life circumstances at 24 months	The conditions of my life are excellent	Five point Likert: Strongly disagree, disagree, neither disagree or agree, agree, strongly agree	4	0.67	0.68	Mean	Increase
Problem alcohol use – AUDIT at 24 months	How often in the past year have you had a feeling of guilt or remorse after drinking?	Variety	10	0.81	0.79	Binary: score of 7/8 cut	Decrease
Quarrelling about alcohol at 24 months	In the past 12 months have you quarreled with any of your female sexual partners about your drinking?	Binary: Yes/No	1			Binary	Decrease
Last sexual partner at 24 months	The last time you had sex was it with a main partner, another partner (khwapeni) or one off partner or ex-partner?	Main partner, casual partner, once-off, ex-partner	1			Binary - main partner versus others	Increase
Transactional sex at 24 months	In the past 12 months, please think about any woman you had sex with just once or any casual partner or khwapheni. Do you think any of them may have become involved with you because they expected you to give or you gave cash or money to be looked after?	Binary: Yes/No	5			Binary: never, compared to once ore more	Decrease
Work shame at 24 months	I am ashamed to see my girlfriend because I don’t have money	Four point Likert: Strongly disagree, disagree, agree, strongly agree	4	0.60	0.57	Mean	Decrease
Work stress at 24 months	I am frequently stressed or depressed because of not having enough work	Four point Likert: Strongly disagree, disagree, agree, strongly agree	4	0.75	0.78	Mean	Decrease
Stealing because of hunger at 24 months	How often in the past 4 weeks have you taken something that was not yours because you did not have enough food or money?	Never, once, two or three times, more often	1			Binary: never stolen versus once or more	Decrease
Mobilisation of cash in emergency at 24 months	If you had an emergency at home and needed R200, how easy would you say it would be to find the money?	Very difficult, somewhat difficult, fairly easy, easy	1			Binary - very difficult and somewhat difficult compared to others	Increase

#### Participant timeline

Recruitment into the study was completed over a 12-month period. All clusters agreed to participate in May–July 2015. The majority of control clusters (*n* = 16) had baseline data collected between October and December 2015. In 2016 just under half the intervention clusters (*n* = 8) had baseline data collected in January to February 2016 and then received the intervention until June 2016. From August to September 2016 remaining clusters (intervention *n* = 9; control *n* = 1) had baseline data collection. Intervention clusters then received the intervention until March 2017. Baseline data collection and intervention delivery took place over an extended period, as recruitment had to be staggered due to intervention staffing availability, and a five-month delay was introduced mid-baseline because of safety considerations related to the build up to the August 2016 local government elections.

**Fig. 1:**
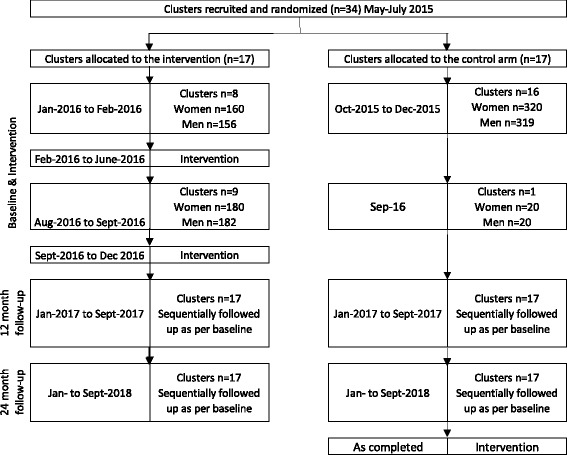
Cluster and Participant timeline

Twelve month post-baseline follow up will be from January to September 2017 with clusters/participants traced sequentially. Similarly, 24 month post-baseline follow up will be January to September 2018. Control arm clusters will receive the delayed intervention following end-line data collection (see Fig. 1).

#### Sample size

In the pilot study past year prevalence of physical and/or sexual IPV perpetration and victimisation was 45% among men and 41% among women. The sample size calculation was modelled on a range of possible baseline rates (see Table 3). The sample size assumes 14 participants will be followed up per cluster (of the 20), but figures are robust to a follow up of only 12 per cluster. It also assumes a coefficient of variation k = 0.2 and modelling to 80% power. The table shows that given the assumption of 14 male (and 14 female) participants per cluster, 16 clusters we will have power to detect a range of possible baseline incidence rates and impact scenarios, and analyse men and women separately. Our power will be further enhanced through multiple assessments (12 and 24 months) and an additional two clusters. The additional two clusters are in case of cluster drop out post-recruitment given the illegality of informal settlements, leading to potential government removal.

**Table 3:** Sample size calculation

IPV incidence (12 months)			# of clusters required by arm
Baseline incidence	Intervention incidence at 24 months	Power	
36	29	80	16
45	36	80	15
41	33	80	16
24	19	80	14
18	14	80	13
30	24	80	15

### Recruitment

#### Clusters

Cluster recruitment occurred between May and June 2015. Project Empower approached ward councillors for permission letters. In the eThekwini context, Ward Councillor permission was critical and implementation could not have happened without it. Throughout the process, Ward Counsellors were made fully aware of what phase the study was at, enabling on-going access and community buy-in. Once secured, local development committees and other key actors were approached for permission to work in specific clusters/informal settlements. Throughout the process of accessing communities the study was described as a project that hopes to help young people improve their lives – as such it did not focus on either the violence or livelihoods aspects of the work.

#### Individual participant community mobilisation and consent processes

Once permission to access clusters was secured and clusters randomised, Project Empower hosted a number of community meetings to brief community members about the trial, its nature and design. During these community meetings, Project Empower requested those eligible and interested in the trial to contact the project staff by sending free ‘please call me’ messages from cell phones. Most young people in informal settlements have access to cell phones. Those who do not can easily access cell phones through low-cost stalls on the side of the road.

Potential participants were invited to meet with project staff in a central location in their community. Spaces varied, including community halls, or a spare room off a shop. Over approximately two days, participants arrived and Project Empower and HEARD staff explained the trial in detail, including the intervention and ethics. Informed consent process only occurred after initial agreement of participants.

### Methods: Assignment of interventions

Randomisation into intervention and control (delayed intervention) arms was undertaken at the cluster level once clusters agreed to participate. Each cluster was allocated a cluster number and randomisation was performed by the study statistician, using a system of numbers. At the time of randomisation the statistician did not know which number was allocated to which cluster. Public randomisation of clusters was not done due to political sensitivities.

Individuals were not blinded to the study arm at time of recruitment and informed consent. While this may lead to recruitment bias, high levels of mistrust around research often found in marginalised communities, and historical experiences of being promised support and then not receiving it were of greater concern for participant wellbeing and project staff safety.

### Methods: Data collection, management and analysis

#### Data collection methods

The study used self-completed questionnaires on cell phones lent to participants. The questionnaire was available in English, isiXhosa or isiZulu. Project staff were available to provide further assistance in terms of how the system worked and with the meaning of questions.

#### Retention of cohort

There are high levels of mobility amongst young people in urban informal settlements and retention in the study is a major concern. A range of strategies to retain the cohort were developed including collection of details of friends and family members of participants in case we lose direct contact, sending SMSes regularly throughout the follow-up and recruiting community members to help identify participants.

In addition, there is an escalating cash incentive for control and intervention arms. Such an approach has been used before to retain cohorts [50]. For the intervention arm the incentive is: baseline – R100 (~US$7); 12 months – R150 (~US$11); 24 months – R300 (~US$21). For the control arm: baseline – R300 (~US$21); 12 months – R450 (~US$31); 24 months – R600 (~US$42).^2^ We set the level unequally between arms as we anticipated some loyalty from the intervention arm due to the engagement in the intervention. We felt we would need to more strongly incentivise control arm retention.

The impact of what could be considered unconditional cash transfers is likely to be minimal. Previous research on cash transfers only show impact when transfers are over several months, or they are of a large value. Additionally, impact has often been shown to be negligible on violence measures or inconsistent. For instance, in Latin America cash transfers over time have shown mixed outcomes. One study of a long-term transfer of US$15 per month (approx. 6–10% of income) showed a marginal increase in controlling behaviours by partners, but no impact on physical or sexual violence [51]. Another of US$40/month over 6 months, showed a reduction in physical and/or sexual violence at end line [52]. Once off cash transfers have needed to be of a high value to show impact, for instance Give Directly in Kenya provided once-off payments of US$1100 direct transfers to households and showed impacts on sexual violence [53]. As such, the incentives are neither of a long-enough duration (i.e. repeat payment every month) nor of enough significant value, to be likely to have impact on the primary or secondary outcomes of the study.

### Data management

As questionnaires are self-completed on cell phones, data management issues and missing data are minimized. Once questionnaires are completed they automatically (assuming 3G connectivity, which is widely available) upload to a secure database hosted by the cellphone/questionnaire provider Mobenzi.

### Statistical methods: Quantitative data analysis

#### Data analysis

The main analysis will be by intention to treat (ITT) and will be done separately for males and females. It will involve a comparison of IPV prevalence between the 2 study arms.

Generalized Linear Mixed Models will be used to compare proportion of women who experienced IPV in the 12 months preceding each follow up data collection point, which will be at 12 months and at 24 months data collection waves [54]. A similar model will be fitted to compare IPV perpetration among men. The study clusters will constitute the random component of the mixed model, while the treatment arm and baseline IPV experience/perpetration status representing the fixed component of the model. Gauss-Hermite quadrature integration method will be to obtain likelihood functions for the observed data [55]. To check for any inaccuracies in the quadrature approximations that may arise due to large intra-class correlations, the number of integration points will be systematically increased so achieve a more accurate approximation of the log-likelihood will be varied. The ordinary Gauss-Hermite quadrature integration method will be compared to the adaptive Gauss-Hermite quadrature method before deciding on the final model. The adaptive Gauss-Hermite quadrature method uses the observed data for each individual cluster, in deriving the maximum likelihood [54].

For outcomes using scales, the score/scale means at 12 months and 24 months will be compared between arms with adjustment for baseline.

### Qualitative process evaluation

Given the limited understanding of how participatory group-based interventions are delivered in ‘real-life’ and that processes of change are often ‘black boxes’ in large trials [41, 56], this study has an integrated qualitative process evaluation. The process evaluation comprises qualitative research with participants (both male and female) and facilitators.

Qualitative research with participants seeks to understand the inter-relationship between gender inequalities, livelihoods and IPV, and how the intervention works to support participants to change, or not. Additionally, a significant focus is on how social contexts shape intervention outcomes. In two clusters (non-randomly selected) fieldworkers will conduct interviews with approximately 40 participants (20 men and 20 women) at baseline and then again at 12 and 18 months post-intervention; participant observation will occur throughout the follow-up period. In one cluster, women will use photovoice [57] to further explore experiences.

Qualitative research with facilitators is focused on understanding how facilitators deliver manualised interventions, the challenges of delivering interventions in informal settlements, and how facilitators work to overcome these challenges [58]. Methods include in-depth interviews and focus-groups with facilitators and observations of two non-randomly selected clusters over the intervention.

### Cost-effectiveness evaluation

To date, economic evaluations of interventions for the prevention of IPV in low and middle income countries (LMICs) are limited [59, 60]. The economic evaluation of Stepping Stones and Creating Futures compares the cost, and impact of the intervention, to determine its cost-effectiveness. The main analysis will calculated the cost per incident of physical and/or sexual IPV averted. Women and men will be analysed separately.

A micro-costing approach from the provider perspective will be applied to the economic evaluation, to identify, quantify and value all resources used to develop and deliver the intervention. For intervention development (from 2011 to 2014) costing will be retrospective, while for intervention delivery in the evaluation it will be prospective. For both phases, interviews with research and intervention staff will be conducted to identify resources and activities. Relevant financial records will then be extracted. Employees’ time will be valued at the salaries they receive. Any resource for which no cost is incurred will be valued at its replacement cost. In addition, a time-and-motion study of facilitators delivering the intervention will be undertaken to determine facilitators’ time allocation across core intervention tasks, administration and research.

Finally, the costing also includes a household/individual perspective, accounting for costs participants incurred to receive the intervention (e.g., transport cost, work opportunities forgone). This will contribute to monetising the burden intervention attendance places on participants, and provide an estimate of how much they value the intervention. Participant costs are collected through paper and pencil self-completed questionnaires during the second-to-last intervention session.

Costs and outcomes will be discounted using a 3% discount factor. Uni- and multi-variable sensitivity analysis will address uncertainty in unit cost estimates, or assumptions regarding resource use or outcomes.

### Ethics

Undertaking research on intimate partner violence has particular challenges. The study is structured in accordance with the ethical principles provided by the World Medical Association Declaration of Helsinki (last updated in 2013), and the Belmont report (1979). Both these documents emphasize respect for person’s autonomy, justice, beneficence, and non-maleficence (do no harm) in the conduct of research with human participants.

Undertaking research on violence against women has particular challenges. As such, specific guidance for conducting this work is provided in the WHO Ethical and Safety Recommendations for Research on Domestic Violence Against Women [61, 62]. This trial somewhat adhered to, but also diverged from, the WHO safety guidelines for research on VAW. The WHO guidelines accentuates the necessity to disguise the violence focus of the research in order to enhance the safety and protection of research participants [63]. These guidelines provide various strategies of protecting research participants. These include interviewing only one woman per household, and not enrolling and interviewing men and women from the same cluster [63]. As argued by R Jewkes, Y Sikweyiya, M Nduna, NJ Shai and K Dunkle [63] this requirement is practicable to implement in survey studies [61], but it was difficult to do so in this intervention trial, particularly because of its design. First, as its primary outcome, this trial aims to prevent IPV, during recruitment of participants it was difficult to conceal the IPV focus. Second, both men and women are enrolled this trial and both will be asked questions on violence. Third, as participants are young women and men from the same cluster, it is likely that some of them will be involved in relationships, and therefore are likely to have an idea of what their partner will be asked.

However, the trial has been designed to ensure that the project does not expose participants to more than minimal risk (more than everyday risk). The study design has measures in place to minimise the potential of harm to participants (e.g. third party retaliation) and to respond to any adverse consequences i.e. emotional or psychological harm that may result from the questions that will be asked in this study. This includes use of self-completed questionnaires and the option to refer participants into supportive care if necessary.

If participants do become distressed during research, or the intervention, the team has referral mechanisms to provide appropriate support. In addition, given the lack, and often inadequacy, of government-provided services, an ‘ethics fund’ has been established. The ethics fund provides short-term, limited relief to participants who have exhausted formal state options for support, and who would be unable to continue their participation in the group without relief. Disbursements are approved following a written application by the Project Empower Programmes Manager outlining the details of the case, and is reviewed by two senior staff and a board member of Project Empower. Relief provided includes access to professional psycho-social services or medical appointments for cases where primary health care clinics have been unable to treat a participant, as well as material relief such as food, or transport. There has been limited use of the fund; examples of recipients to date include providing blankets to a participant whose bedding was lost during flooding, and the purchase of prescription medication for another.

No data monitoring board (DMB) was feasible for this study. The entire intervention is provided to participants before the follow-up data collection occurs, as such assessment of harms cannot happen until the intervention is completed.

The study was approved by the Biomedical Research Ethics Committee (BREC) at the University of KwaZulu-Natal, Durban, South Africa (BFC043/15) and the South African Medical Research Council Ethics Committee (EC006–2/2015).

#### Consent

All participants were provided with, and signed informed consent forms, in a language of their choice, with verbal explanation as well. They have the option to withdraw throughout the study without detrimental outcomes to them.

#### Confidentiality

The trial includes questions about participation in illegal activities. To ensure confidentiality all questionnaires are self-administered on cell phones. Participants were allocated a unique study code, not linked to their names, and thus their responses will not be traceable back to them. Using cell phones will allow us to collect data anonymously and this will therefore protect researchers and participants should the latter disclose illegal activities or practices in the interviews.

However, there are limits to confidentiality, particularly during the intervention where there may be risks of participants disclosing information which may require reporting. While this is unlikely to happen, participants have been alerted in the informed consent, and facilitators have been trained to stop participants before they reveal issues that may need to be reported. However, unsolicited reports may emerge [64] and, if participants do report such activities to fieldworkers, facilitators or team members, they may have to report onwards to ethics committees and potentially the police.

## Results

### Cluster recruitment

All clusters approached for participation were willing to be included in the study. During the cluster recruitment, a local government election was held and in number of clusters new Ward Councillors were elected. The cluster’s continued participation in the study was reconfirmed by the new Ward Councillors. In total 34 clusters were recruited.

### Comparison of baseline results between arms

A total of 680 women and 677 men were enrolled into the study. Table 2 shows the Cronbach’s alphas for the scales used in the study separately for women and men. All scales exhibit similar levels of moderate to good internal consistency across the two groups.

Table 4 shows the social and demographic characteristics of participants recruited in each arm at the individual level, adjusting for the clustered nature of data. Women and men were both relatively young, with everyone falling into the target range of 18–30. There was balance between arms, with the mean age of women in the control arm being 23.7 years (SD3.43) and 23.6 years (SD3.28) in the intervention, with men very similarly aged in the control arm at 23.5 years (SD3.41) and intervention arm 23.8 years (SD3.27).

**Table 4:** Baseline socio-demographics of study participants

			Women						Men				
			Controls(*N* = 340)		Intervention(*N* = 340)				Controls(*N* = 339)		Intervention(*N* = 338)		
		N	n/mean	%/SD	n	%	*P* value	N	n	%	n	%	*P* value
Age	mean/stdv	680	23.7	3.43	23.6	3.28	0.597	677	23.5	3.41	23.8	3.27	0.244
Relationship status:	none	126	68	20.0%	58	17.1%	0.572	145	65	19.2%	80	23.7%	0.006
	Living with partner	113	57	16.8%	56	16.5%		73	49	14.2%	24	7.4%	
	Not living with partner	441	215	63.2%	226	66.5%		459	225	66.7%	234	68.9%	
Education	Primary	55	31	9.1%	24	7.1%	0.068	77	36	10.6%	41	12.1%	0.742
	Secondary	419	195	57.4%	224	65.9%		393	201	59.3%	192	56.8%	
	Matric	206	114	33.5%	92	27.1%		207	102	30.1%	105	31.1%	
Currently studying	No	627	313	92.1%	314	92.4%	0.886	592	291	85.8%	301	89.1%	0.206
	Yes	53	27	7.9%	26	7.7%		85	48	14.2%	37	11.0%	
Food security	None or little	127	43	12.7%	84	24.7%	0.0003	125	55	16.3%	70	20.7%	0.256
	Moderate	342	187	55.0%	155	45.6%		382	200	59.2%	182	53.9%	
	Severe	211	110	32.3%	101	29.7%		169	83	24.5%	86	25.4%	

In terms of relationships there were no significant differences amongst women by study arm, with about one fifth of women reporting not currently being in a relationship (20.0% control, 17.1% intervention), and a similar proportion reporting living with a boyfriend (16.8% control, 16.5% intervention), while the majority had a boyfriend they did not live with (63.2% control, 66.5% intervention). Amongst men there was a significant difference in relationship status (*p* = 0.006). The main difference was that men in the control arm were more likely to report living with a girlfriend (14.2% control, 7.4% intervention), other types of relationship were similar by arm, and most men (66.7% control, 68.9% intervention) had a girlfriend but were not cohabiting.

Education levels were low amongst participants and balanced across arms. Overall less than a third of women and men had completed matric/Grade 12 (school leaving certificate), but most had some secondary school education (57.4% control, 65.9% intervention). Few women and men reported currently studying, with similar proportions in both arms for women (7.9% control, 7.7% intervention) and men (14.2% control, 11.0% intervention).

Young women and men experienced high levels of food-insecurity as assessed by the Household Hunger Scale [65]. Overall the proportion experiencing severe food insecurity was a quarter for men and a little higher for women, and was very similar for men and women between the arms. There were differences among women by arm in experience of moderate food insecurity, which was reported by just over half (55.0%) of control arm women compared to 45.6% intervention arm.

## Discussion

Overall baseline data suggests balance between arms on most socio-demographic measures. This suggests that, while participants were not blinded to study arm at recruitment, this did not affect who was recruited in terms of observable characteristics. Baseline socio-demographic data also highlight the high levels of food insecurity and low educational levels of participants entering the study. These challenges shape young people’s experiences of relationships and job seeking, and emphasise the need for combination interventions to reduce poverty and strengthen socio-economic outcomes, irrespective of wider aims of IPV prevention.

The study has a number of limitations. The evaluation may show an under-estimation of effect of the intervention. While the potential for contamination is limited as clusters are discreet and the intervention is intensive in nature, there is mobility in informal settlements that may lead to sharing of information, as well as loss to follow up. Of concern is the wide-scale implementation of the DREAMS programme (Determined, Resilient, Empowered, AIDS-free, Mentored, and Safe women) funded by PEPFAR, Gates Foundation and Girl Effect, and additionally Global Fund programming in the eThekwini Municipality, including study communities. Both programmes include aspects of gender transformation and draw heavily on Stepping Stones, and Stepping Stones and Creating Futures, as well as other approaches to reduce HIV-vulnerability and prevent IPV, with the potential that control arm participants receive the intervention, though they were randomized into the delayed intervention. In addition, participants were not blinded to study arm at time of recruitment. While this was done for practical and political realities, and there were few differences between arms in terms of socio-demographic characteristics, there may be unmeasured bias between arms not reflected in the data. Going forward, participants will not be blind to their intervention group and this may impact on self-reported behaviours. Unfortunately, we have no way of knowing this, but the fairly large number of trials with negative findings in this research field overall suggests that invitation to participate in an intervention of the type we are researching does not overwhelmingly bias responses.

While there is growing evidence of the importance of gender transformative and economic strengthening interventions to reduce women’s experiences of IPV, the number of large well evaluated interventions remains limited [21]. Research in South Africa on IPV prevention interventions has predominantly been conducted in rural areas and so little is known about the effectiveness of interventions in very challenging urban settings [25]. Rigorously evaluated interventions directly assessing the impact of including men in such interventions are very limited and the impact of economic empowerment on men’s use of violence is particularly unclear [25]. As such, the Stepping Stones and Creating Futures evaluation is important and will considerably increase knowledge of what works to prevent violence against women in South Africa. The qualitative evaluation will enable processes of change to be understood and to capture unforeseen impacts, in addition the economic evaluation enables the cost-effectiveness of the intervention to be assessed. The multi-pronged nature of the evaluation will also contribute to strengthening future interventions in the field of IPV prevention.

## Additional file

Additional file 1: Completed SPIRIT Guidelines for the Stepping Stones and Creating Futures Trial. (DOC 125 kb)

## Footnotes

1 See: www.whatworks.co.za

2 Dollar rate calculated on 14 September 2016, approximately R7 to US$1
